# Practical Observations and Suggestions on Medicine

**Published:** 1845-04

**Authors:** 


ART. VIII.—*Practical Observations and Suggestions in Medicine.* By MARSHALL HALL, M.D. F.R.S.L. & E., &c. &c.—*London*, 1845. 8vo, pp. 359.

IT is with unfeigned concern we place our notice of this book in the Second Part of the Journal. A work from the pen of the most original and distinguished physiologist of the day, on whatever subject written, ought to command a detailed review. We are, however, compelled to state, after careful perusal, that the present volume neither demands nor justifies such a mode of treatment. Although it consists of more than fifty chapters, it contains very little that is novel, and still less that is valuable. Many of the papers have already appeared in the Journals; others are repetitions from the author's former publications; and several are supplied by his friends. Not a few of the practical novelties seem to us of doubtful soundness, and some excite surprise as coming from a man of science and long experience. In one word, we think the present work altogether unworthy the acknowledged great talents and high reputation of its author; and we think he has been very ill-advised in bringing it before the profession in its present form. It looks as if its contents had been carelessly huddled together, and the good and the bad sent to the printer, on a sudden impulse to make a book. A proceeding of this kind might be overlooked in a man of no reputation; but Dr. Hall ought to have greater respect for his own fame, than to risk the consequences of such a step. He cannot afford to bring out an inferior work. It cannot lie hid as the production of a man unknown. Like the author's former works, it will be read by the whole medical profession; and if it cannot obscure the brightness that preceded it, it will at least look darker from the contrast. We write these words in sorrow not in anger; and in writing them, we feel that we are taking the part of Dr. Hall against himself. He has written good books; he can write good books; he ought to write good books; and his disappointed readers throughout the world must be reminded that if on the present occasion he has not done so, it is because he has not chosen to do so.

